# *PTEN* hamartoma tumour syndrome: case report based on data from the Iranian hereditary colorectal cancer registry and literature review

**DOI:** 10.1186/s13000-023-01331-x

**Published:** 2023-04-04

**Authors:** Zahra Rahmatinejad, Ladan Goshayeshi, Robert Bergquist, Lena Goshayeshi, Amin Golabpour, Benyamin Hoseini

**Affiliations:** 1 Department of Medical Informatics, Faculty of Medicine, Mashhad University of Medical Sciences, Mashhad, Iran; 2 Department of Gastroenterology and Hepatology, Faculty of Medicine, Mashhad University of Medical Sciences, Mashhad, Iran; 3 Surgical Oncology Research Center, Mashhad University of Medical Sciences, Mashhad, Iran; 4 Ingerod, Brastad, SE-454 94 Sweden; 5 Formerly UNICEF/UNDP/World Bank/WHO Special Programme for Research and Training in Tropical Diseases (TDR), World Health Organization, Geneva, Switzerland; 6 School of Allied Medical Sciences, Shahroud University of Medical Sciences, Shahroud, Iran; 7 Pharmaceutical Research Center, Mashhad University of Medical Sciences, Mashhad, Iran

**Keywords:** Cowden syndrome, PHTS, *PTEN* hamartoma, Early diagnosis, Colorectal polyposis, Case reports, Case series

## Abstract

**Background:**

*PTEN* hamartoma tumour syndrome (PHTS) is a rare hereditary disorder caused by germline pathogenic mutations in the *PTEN* gene. This study presents a case of PHTS referred for genetic evaluation due to multiple polyps in the rectosigmoid area, and provides a literature review of PHTS case reports published between March 2010 and March 2022.

**Case presentation:**

A 39-year-old Iranian female with a family history of gastric cancer in a first-degree relative presented with minimal bright red blood per rectum and resistant dyspepsia. Colonoscopy revealed the presence of over 20 polyps in the rectosigmoid area, while the rest of the colon appeared normal. Further upper endoscopy showed multiple small polyps in the stomach and duodenum, leading to a referral for genetic evaluation of hereditary colorectal polyposis. Whole-exome sequencing led to a PHTS diagnosis, even though the patient displayed no clinical or skin symptoms of the condition. Further screenings identified early-stage breast cancer and benign thyroid nodules through mammography and thyroid ultrasound.

**Method and results of literature review:**

A search of PubMed using the search terms “Hamartoma syndrome, Multiple” [Mesh] AND “case report” OR “case series” yielded 43 case reports, predominantly in women with a median age of 39 years. The literature suggests that patients with PHTS often have a family history of breast, thyroid and endometrial neoplasms along with pathogenic variants in the *PTEN*/*MMAC1* gene. Gastrointestinal polyps are one of the most common signs reported in the literature, and the presence of acral keratosis, trichilemmomas and mucocutaneous papillomas are pathognomonic characteristics of PHTS.

**Conclusion:**

When a patient presents with more than 20 rectosigmoid polyps, PHTS should be considered. In such cases, it is recommended to conduct further investigations to identify other potential manifestations and the phenotype of PHTS. Women with PHTS should undergo annual mammography and magnetic resonance testing for breast cancer screening from the age of 30, in addition to annual transvaginal ultrasounds and blind suction endometrial biopsies.

## Introduction

*PTEN* hamartoma tumour syndrome (PHTS), commonly defined as multiple hamartoma syndrome, is a rare autosomal genodermatosis with a heterogeneous phenotype that is clinically characterized by numerous hamartomas of ectodermic, mesodermic or endodermic origin with an elevated lifetime risk of developing endometrial, breast, thyroidal, colorectal or renal carcinomas [1]. PHTS is commonly (80% of all cases) associated with pathogenic variants affecting the phosphatase and tensin homologue (*PTEN*) gene [2, 3]. Other disorders caused by dysfunction of this gene are Bannayan-Riley-Ruvalcaba syndrome (BRRS) and Cowden syndrome (CS) [4]. BRRS tends to affect children, while CS are most commonly seen in adults. PHTS is primarily caused by pathogenic gene mutations (variants) in the *PTEN* tumour suppressor gene [5].

The estimated incidence of PHTS is around one in 200,000 people [1, 6], but this is likely to be an underestimation due to its phenotypic diversity and difficulty in recognition. Consequently, PHTS poses a dilemma for clinicians, who must conduct multiple medical evaluations of affected patients before the diagnosis is reached [7]. Early detection is crucial, as the best potential prognosis for patients with PHTS rests on accurate clinical observation plus ongoing surveillance of affected individuals [2]. The diagnosis is primarily clinical with genetic follow-up, and the National Comprehensive Cancer Network (NCCN®) annually reviews and develops the diagnostic criteria created by Eng et al. [8–11].

Clinical guidelines for diagnosis and surveillance are needed for PHTS due to its diversity and infrequency. Reporting cases with different characteristics can help improve national and international approaches to early diagnosis of affected patients and their family members, who are at increased risk of developing several cancers in their lifetime. Previous case reports have highlighted clinical or skin symptoms associated with PHTS, such as papillomatous skin lesions, macrocephaly, gingival hypertrophy and blood vessel problems [12–19]. CS-related hamartoma polyps in various parts of the body have been reported [6, 17], including a 16-year-old Iranian female with pathognomonic cutaneous features of CS, who was evaluated for the *PTEN* gene through testing by the polymerase chain reaction (PCR) [19]. However, there are no reports from Iran of breast cancer associated with PHTS.

The Iranian Hereditary Colorectal Cancer Registry (IHCCR) is a programme specifically designed to identify individuals at high risk of hereditary colorectal cancer or polyposis in Iran [20–23]. IHCCR confirms these cases through whole-exome sequencing (WES), a comprehensive genetic test that sequences all of the protein-coding gene regions [24]. A 39-year-old female with multiple rectosigmoid polyps, who underwent WES to confirm hereditary colorectal polyposis, was unexpectedly diagnosed with PHTS despite absence of any clinical symptoms of the condition. Here, we summarize the disease manifestations, treatment and management of this case. Furthermore, we conducted a literature review of case reports on PHTS, with the secondary objective to compare the clinical signs found with those reported in other relevant cases.

## Case presentation

A 39-year-old Iranian woman presented with minimal bright red blood per rectum at Emam Reza Hospital in Mashhad, the referral university hospital in north-eastern Iran. The patient was married, unemployed, had nine siblings and denied any history of alcohol, tobacco or medication. The patient presented with multiple skin tags on her neck, which were determined to be non-PHTS-related. An intraoral examination revealed no significant findings. However, early-stage breast cancer had been detected during mammography screening.

**Fig. 1:**
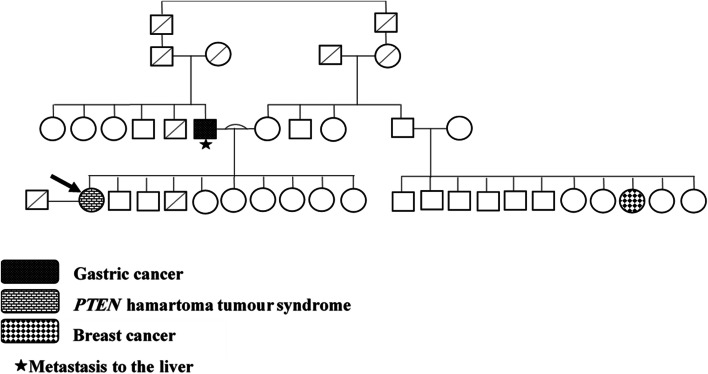
Family tree of the patient, with squares representing men and circles women. Arrow indicates the patient

Notably, the patient had a significant family history of cancer. At the age of 52, her father was diagnosed with cancer of the stomach, which subsequently metastasized to the liver and ultimately led to his death, and a cousin had been diagnosed with breast cancer. There was no reported history of radiation exposure or goitre in her family. The patient’s family tree (Fig. 1) represents all close relatives, both affected and unaffected by disease.

### Diagnostic investigation

Upper endoscopy and colonoscopy were recommended due to resistant dyspepsia, rectal bleeding, family history of stomach cancer and mild anaemia. Oesophago-gastro-duodenoscopy (EGD) revealed 20–50 small sessile polyps in the stomach and duodenum. Microscopic examination of a biopsy from the gastric mucosa showed non-dysplastic, polypoid tissue. Notably, colonoscopy revealed over 50 diminutive sessile polyps in the rectosigmoid area, while other parts of the colon were normal. Subsequent histological examination confirmed these polyps as hyperplastic, hamartomatous polyps.

Since the guidelines issued by the American College of Medical Genetics and Genomics (ACMG) [25] recommend genetic testing for patients with more than 20 colon polyps, the patient was enrolled in the IHCCR programme for genetic consultation. Its charge is to identify individuals at high risk of hereditary colorectal cancer or polyposis in Iran confirming their findings by WES.

#### Whole exome sequencing

DNA was extracted from whole blood using standard procedures. Human whole exome enrichment was performed using ‘Agilent SureSelect V6 Target Enrichment Kit’ (www.agilent.com) according to the manufacturer’s protocol. Briefly, genomic DNA was captured using biotinylated RNA probes, which target all exonic regions and 10 flanking base-pairs (bp). After amplification and sequencing using the Illumina HiSeq4000 platform (Illumina, Inc.), the data were analysed using standard bioinformatics tools. Variant calling was performed using the genome analysis toolkit (GATK) software (https://gatk.broading.org) [26] that detects variations, such as single point mutations and small Indels (within 20 bp). The DNA sequence was mapped and analyzed in comparison with the published human genome build (UCSC hg19 reference sequence). Variants with a minor allele frequency (MAF) ≥ 0.1% (heterozygous variants) or ≥ 1% (homozygous variants) were excluded using 1000 Genomes (Asian), Iranom and the Genome Aggregation Database (gnomAD) [27]. Sorting intolerant from tolerant (SIFT) [27], Polymorphism Phenotyping, version 2 (PolyPhen2) combined with HumVar, a dataset that provides pre-computed predictions of the functional impact of human non-synonymous (change of amino acids) variants [28] and combined annotation-dependent depletion (CADD) with Phred score ≥ 20 [29] were used for prediction of missense variants. The raw data used in the genetic evaluation of this case are provided as a supplementary file [30].

The WES analysis of the patient’s DNA revealed a heterozygous pathogenic variant on the *PTEN* gene, specifically a pathogenic non-sense variant (c.697C > T, p.Arg233Ter) with a CADD score of 37 and a Deep Neural Network (DANN) [31] score of 0.997 (Table 1). The *PTEN* gene is associated with autosomal dominant PHTS [32], but in this case, the patient did not exhibit any skin or clinical symptoms of the condition. According to guidelines of the National Comprehensive Cancer Network (NCCN) and the European Reference Network on Genetic Tumour Risk Syndromes (ERN GENTURIS), PHTS-suspected cases should undergo gene panel testing [9, 33].

**Table 1:** Details on the methods and results of whole exome sequencing in the study

**ID**	**Germline candidate gene**	**HGVS.c**	**HGVS.p**	**Coding Impact**	**gnomAD**	**SIFT**	**PolyPhen2**	**DANN**	**CADD**	**ClinVar database**	**ACMG classification**
P9	*PTEN*	c.C697T	p.R233X rs121909219	Non- sense	-	-	-	0.997	37	Pathogenic	Pathogenic

The table follows the nomenclature recommended by the Human Genome Variation Society (HKVC) where HGVS.c refers to the coding variant and HGVS.p to the protein level variant

*gnomAD* genome aggregation database (provision of allele frequency), *SIFT* Sorting intolerant from tolerant, *PolyPhen2* Polymorphism Phenotyping, version 2, *CADD* Combined annotation dependent depletion (provision of Phred score, version 1.6), *DANN* Deep neural network (provision of a functional prediction score), *ClinVar* Archive of interpretations of clinically relevant variants, *ACMG* American College of medical Genetics and genomics

#### Clinical presentation

The International Cowden Syndrome Consortium (ICSC) [9, 10] recommends surveillance for additional manifestations of PHTS and potentially related malignancies, as outlined in Table 2. Patients suspected of having PHTS are at increased risk of cancers of the breast, thyroid, colon and rectum necessitating further investigation [34]. Furthermore, the American Gastroenterology Association recommends annual comprehensive physical examination, thyroid ultrasound screening beginning at the time of diagnosis, endometrial suction biopsy starting at the age 30–35 and colonoscopy screening beginning at 35 years of age or 5–10 years before the initial documented case of colon cancer in the family [35].

**Table 2:** Diagnostic criteria proposed by the International Cowden Syndrome Consortium [9, 10]

**Pathognomonic lesions highly specific for the Cowden syndrome (CS) include:** • Six or more facial papules (with at least 3 trichilemmomas) • Facial cutaneous papules with papillomatosis of the oral mucosa • Papillomatosis of the oral mucosa with acral keratosis • Six or more palmoplantar patches of keratosis Diagnosis of CS requires at least one of these pathognomonic lesions
**Major criteria less specific for CS but are associated with a higher risk of cancer include:** • Breast carcinoma • Thyroid carcinoma • Macrocephaly (greater than 97%) • Lhermitte-Duclos disease (LDD) • Endometrial carcinoma Diagnosis of CS requires at least two major criteria, with one of them being macrocephaly or LDD
**Minor criteria even less specific for CS but still commonly seen in affected individuals include:** • Thyroid lesions (other than carcinoma) • Learning difficulties or delayed development • Gastrointestinal hamartomas • Lipomas • Fibromas • Fibrocystic disease of the breast • Genitourinary malformations or carcinoma Diagnosis of CS requires at least four minor criteria **Additionally**, **one major criterion and three minor criteria may also indicate a diagnosis of CS**

The patient in question presented with a solid mass measuring 22 × 17 mm in the lower outer quadrant of the right breast, which was detected by mammography. Ultrasonography confirmed the presence of dense, oval nodules with well-defined margins measuring approximately 20 × 15 mm located at the 6 o’clock position of the right breast, 5 cm from the nipple. Based on the Breast Imaging Reporting and Data System (BIRADS), these nodules were categorized as BIRADS IV, indicating a suspicious abnormality that necessitates further workup to ascertain whether or not likelihood of malignancy.

In addition, thyroid ultrasonography revealed multiple, well-defined, isoechoic and hyperechoic nodules in both thyroid lobes. Further evaluation through fine-needle aspiration (FNA) biopsy showed a follicular lesion with undetermined significance classified as BETHESDA III. This category denotes that the specimen obtained is non-diagnostically relevant but does not exclude the possibility of malignancy, with and clinical correlation, repeat FNA and/or surgical excision recommended.

The patient underwent further evaluation as recommended but a biopsy of the endometrium revealed no abnormalities. Biochemical assessments of renal, liver and thyroid function were within the normal range, and the patient did not exhibit macrocephaly, which is a common PHTS feature.

#### Histology and immunohistochemistry

**Fig. 2:**
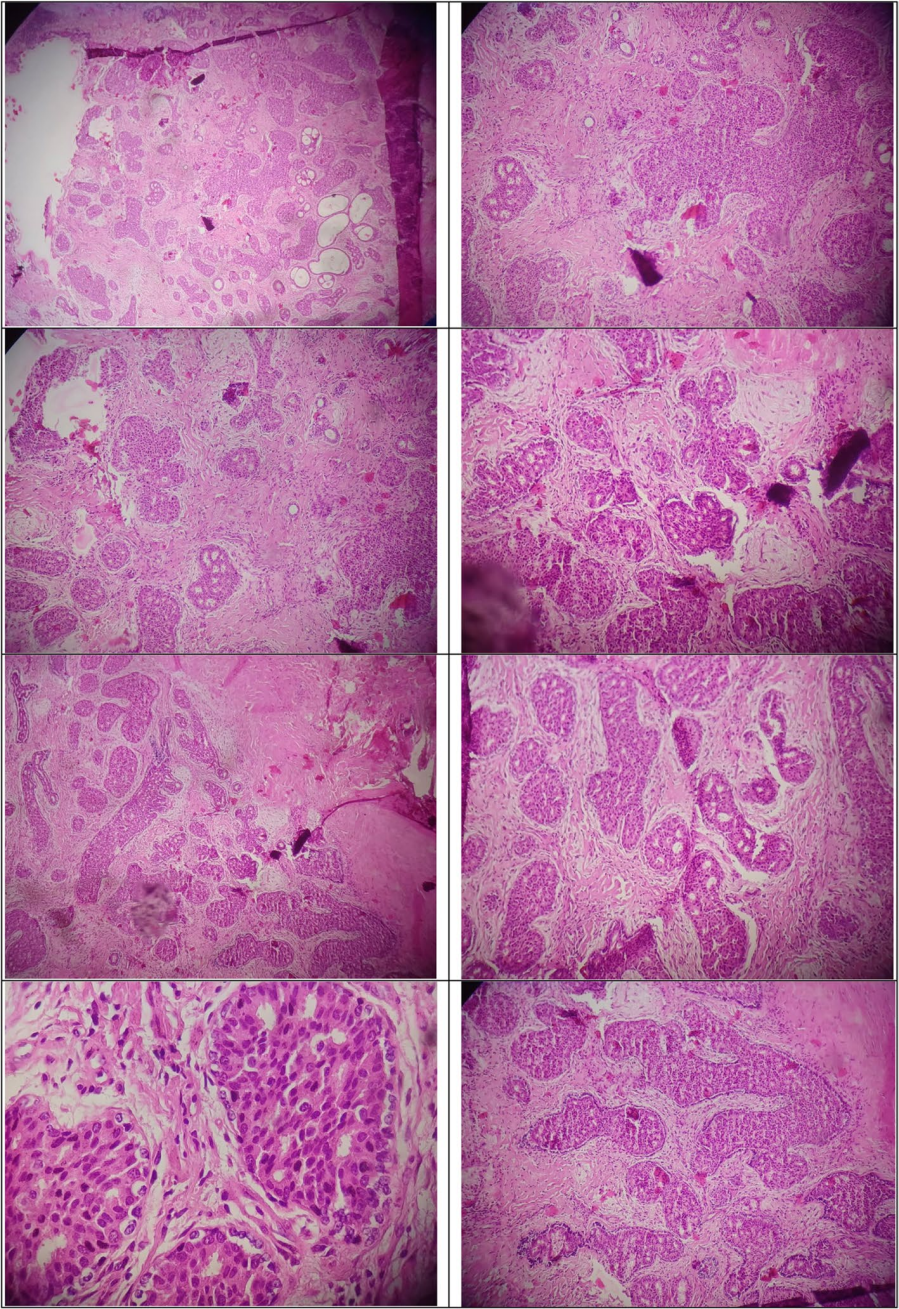
Haematoxylin and eosin staining of breast tissue from the patient (magnification: 100 x); the slides show invasive neoplastic proliferation of atypical epithelial cells with cribriform pattern and desmoplastic stroma consistent with the characteristic features of breast cancer in patients with the *PTEN* hamartoma tumour syndrome

Multiple foci of intraductal hyperplasia with mild to moderate nuclear grade and an area of invasive pattern suspicious for cribriform carcinoma were observed in the breast core needle biopsy (Fig. 2). Immunohistochemistry (IHC) staining according to the 2013 ASCO/CAP *HER2* guidelines [36] was performed and revealed positivity for p63 and actin, indicating a solid nest of ductal carcinoma *in situ* (DCIS). The tumour cells also tested positive for estrogen and progesterone receptors in 90% of the cells. The *HER2* status of the tumour was initially equivocal (2 +), with weak membranous staining in 30% of tumoural cells. To clarify the result, fluorescence *in situ* hybridization (FISH) testing was performed and it revealed a negative *HER2/neu* gene status in the tumoural cells. As a result, the final *HER2* status of the tumour was negative. A Ki-67 index was found in 5% of the tumour cells, indicating a low proliferation rate. Following partial mastectomy, a 2.2 cm invasive cribriform carcinoma with a Bloom-Richardson grade [37] of 1(2 + 1 + 1) was observed. Notably, there was no evidence of vascular or perineural invasion and the surgical margins were free of tumour involvement.

### Literature review

A literature review was conducted using the PubMed database to identify cases of PHTS reported between March 2010 and March 2022. The search strategy used the terms “Hamartoma Syndrome, Multiple” [Mesh] AND “case report” OR “case series”. A comprehensive reference list search of related literature was performed, with the review limited to articles in the English language, excluding letters to the editor and review articles.

**Fig. 3:**
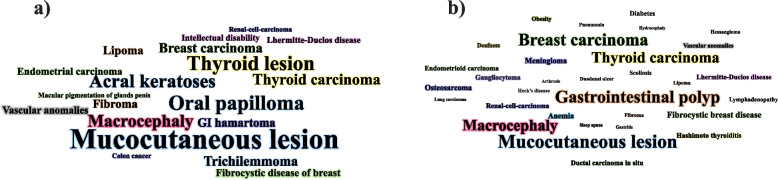
**a** Clinical criteria considered for diagnosing PHTS patients; and **b** Medical observations reported for these cases according to the literature review

**Fig. 4:**
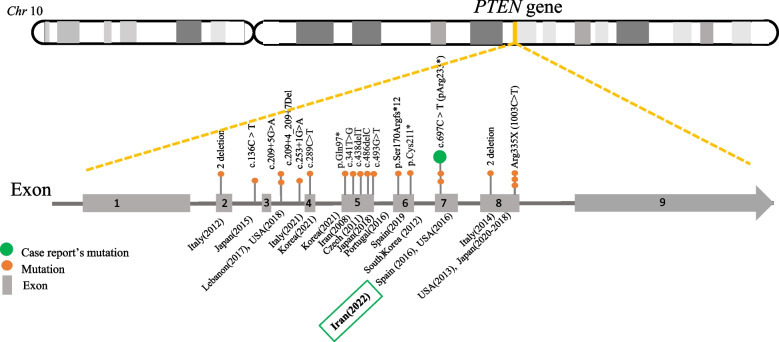
*PTEN* pathogenic variants found in tumours and PHTSs in the case reports reviewed; mutated sites of the pathogenic variants are shown in the exons and introns of the *PTEN* gene

We identified 43 cases of PHTS, the characteristics of whom are summarised in Table 3. The median age of the cases was 39 years (IQR: 31–52; min–max: 14–75) and we noted that the disease predominantly affected women. Of the 43 cases, 23 had a family history of PHTS with complications, such as breast, thyroidal and endometrial neoplasms along with pathogenic variants in the *PTEN/MMAC1* gene. Pathognomonic characteristics of PHTS, including acral keratosis, trichilemmomas, and mucocutaneous papillomas were reported in about 70% of the cases. Figure 3 depicts the clinical criteria used to diagnose PHTS patients and their medical history, as reported in the literature review. Gastrointestinal polyps were frequently reported as clinical manifestations. Figure 4 visualizes the mutated sites of the *PTEN* pathogenic variants indicating that pathogenic variants in exon 5 were frequent.

**Table 3:** Results of the literature review

**Country** **(Year)** **[Ref]**	**Age**	**Sex**		**Chief** **complaints**^a^		**Major criteria**^b^		**Minor criteria**^c^		**History and other clinical manifestations**^d^	**Diagnostic measures**^e^
USA (2012) [38]	42	M		Rectal bleeding		Mucocutaneous lesions, oral papules, acral keratosis, trichilemmoma		Colon cancer Lipomas		**Family history:** Negative	Colonoscopy, genetic test, histology
										**Surgical history:** Colectomy	
										**Medical observations:** Colonic ganglioneuromatous polyps, mucocutaneous features	
	**Laboratory results & detailed information**^f^: **Colonoscopy & microscopic examination:** ganglioneuromatous polyposis**,** specimen contained an adenomatous polyp in addition to multiple, finger-like polyps and mesenteric lipomas; **Genetic test:** pathologic non-sense mutation at the *PTEN* locus										
Spain (2016) [2]	57	F		Dysphagia & recurrent endometrial carcinoma		Breast carcinoma, mucocutaneous lesions, oral papules, acral keratosis, trichilemmoma		Thyroid lesions		**Family history:** NA	Brain and chest CT, MRI of spine, genetic test
										**Surgical history:** Thyroidectomy, hysterectomy	
										**Medical observations:** Uterine leiomyomas, rectal bleeding secondary, anal polyp, sphenoid wing meningioma, meningiomas, thin-walled lung cyst, thoracolumbar scoliosis	
	**Laboratory results & detailed information** **CT:** left sphenoid wing meningioma with extra-conal extension and resultant left optic nerve impingement and proptosis; right breast lesions **MRI:** thoracolumbar scoliosis with spondylosis and multilevel disc disease; **Genetic test:** pathogenic variants in the *PTEN* gene: (c.697C > T) (pArg233*)										
Tunisia (2012) [6]	30	F		Altered bowel habits		Macrocephaly, thyroid carcinoma, mucocutaneous lesions, acral keratosis		FDB, thyroid lesion, gastrointestinal hamartomas		**Family history:** Negative	Endoscopy, colonoscopy, breast US
										**Surgical observations:** Surgical excision of breast nodules & thyroidectomy	
										**Medical observations:** Congenital mental retardation. breast nodules, multiple gastrointestinal polyps, high-arched palate, adenoid facies, kyphoscoliosis and pectus excavatum, iron deficiency anemia	
	**Laboratory result & detailed information** **Breast US:** bilateral nodules compatible with fibrocystic disease; **Endoscopy:** esophageal, duodenal, and gastric sessile polyps; **Colonoscopy**: multiple lesions in the sigmoid and rectum										
USA (2013) [14]	14	F		Menorrhagia refractory to intravenous estrogen		Endometrial carcinoma, Macrocephaly, mucocutaneous lesions, oral papules, trichilemmoma		Lipomas, fibroma		**Family history**: Hashimoto’s thyroiditis in two sisters, thyroid cancer in her father, goiter & fibrocystic breast disease in her mother	Endometrial curettage & pathological examination, PET/MRI of ovaries, colonoscopy, genetic test
										**Surgical history:** Hysterectomy and laparoscopic excision of ovary at age 19 years	
										**Medical observations:** Macrocephaly, endometriosis adenocarcinoma, colon polyps, gum fibroids, benign squamous cyst of right ovary	
	**Laboratory result & detailed information** **Endometrial curettage & Pathological examination:** focal grade 1 endometriosis adenocarcinoma; **PET/MRI:** concerning for significant myometrium invasion, showed no involvement of the ovaries at age 14; **Colonoscopy:** multiple polyposis lesions in transverse and sigmoid colon; **Genetic test:** Arg335X (1003C > T) mutation in the *PTEN* gene										
Brazil (2015) [13]	36	F		Gingival growth		Macrocephaly, thyroid carcinoma, mucocutaneous lesions, oral papules		NA		**Family history**: NA	Histopathologic evaluation
										**Surgical history:** Thyroidectomy, removing papules from hand & stomach	
										**Medical observations:** Thyroid carcinoma, macrocephaly, Hashimoto thyroiditis & gastritis, arthrosis & fibromyalgia, depression, breast nodules, Heck’s disease, ductal ectasia	
	**Laboratory result & detailed information** **histopathologic evaluation:** epithelial hyperplasia as a histological finding of biopsy from the gingival nodule										
Croatia (2019) [18]	39	F		White spot on gingiva, face, & hand		Thyroid carcinoma (follicular), mucocutaneous lesions, oral papules, acral keratosis, trichilemmoma		FDB, Lipoma, Fibroma, Vascular anomalies		**Family history**: Endometrial polyp in mother & thyroid cancer in sister	Gynecological US, cardiological examination
										**Surgical history:** Surgery of thyroid, cavernous ovary lymphangioma and leiomyomas of uterus	
										**Medical observations:** Fibrocystic changes breast papillomatosis, liver hemangioma, lipoma, multiple fibromas, genitourinary tumours, endometrium polyp, myomas, thyroid abnormalities, pericardial effusion	
	**Laboratory result & detailed information** **Gynecological US:** polyp in the endometrium and multiple myomas of the myometrium; **Cardiological examination:** pericardial effusion										
USA (2017) [39]	63	M		Lesions on calf,abdomen, clavicle & elbow		Thyroid carcinoma, mucocutaneous lesions, oral papules, acral keratosis, trichilemmoma		Thyroid lesion		**Family history:** Unremarkable	CT-scan of neck, IHC
										**Surgical history:** Thyroidectomy, cholecystectomy	
										**Medical observations:** Thyroid cancer, hypertension, obstructive sleep apnea, nodular lesions in right parotid gland, SLA, CCA	
	**Laboratory result & detailed information** **CT:** Nodular lesions in the right parotid gland; **IHC:** Sebaceous lymph adenoma, Clear Cell Acanthoma,										
Czech (2011) [40]	55	F		Epileptic paroxysms		Macrocephaly, thyroid carcinoma, mucocutaneous lesions		Intellectual disability, Thyroid lesion, GI hamartomas		**Family history:** colon cancer in mother	Endoscopy, brain CT, IHC, genetic test
										**Surgical history:** thyroidectomy, hysterectomy, adnexectomy and abdominoperineal surgery	
										**Medical observations:** Gastric cancer, Myomatosis, Adenopapilocarcinoma of ovary, epileptic paroxysm, decline in cognitive functions & memory, polyps in stomach, duodenum & colon	
	**Laboratory result & detailed information** **Endoscopy**: hundreds of polyps in the stomach, duodenum and colon; **CT**: frontal lesion; **IHC**: invasive follicular carcinoma benign meningioma; **Genetic test**: heterozygous deletion mutation at (c.438delT), exon 5										
Japan (2020) [41]	47	F		Tumour in her right breast		Breast carcinoma, thyroid carcinoma, LDD		Thyroid lesions		**Family history:** Breast cancer in her mother and sister	Breast US, pathological examination, chest CT, chest & brain MRI
										**Surgical history:** Mastectomy, thyroidectomy	
										**Medical observations:** Thyroid carcinoma, breast carcinoma, LDD	
	**Laboratory result & detailed information** **Breast US:** irregular marginated hypoechoic mass measuring 15.1 × 15 × 9.4 mm in the 12 o’clock region of the right breast; **Pathological examination:** ductal carcinoma in situ (DCIS) in the right mammary gland and invasive ductal carcinoma in the left mammary gland; **Chest MRI:** Primary tumour of the right mammary gland to be a mass 15 mm and early phase linear enhancement for the left side = > stage 0 (cTisN0M0) right breast cancer and stage IIA (cT2N0M0) for the left side; **Brain MRI:** alternative isointense and hyper intense bands in the left cerebellar hemisphere; **Chest CT**: no lymph node metastases or distant metastases										
Portugal (2020) [42]	28	F		NA		Macrocephaly, thyroid carcinoma, breast carcinoma, Mucocutaneous lesions: acral keratosis		Intellectual disability		**Family history:** thyroid carcinoma in her mother and aunt	Breast CT & IHC, thyroid & breast US, genetic test
										**Surgical history:** mastectomy, thyroidectomy	
										**Medical observations:** Hydrocephaly, severe microcytic & hypochromic anemia, macrocephaly, thyroid & breast cancer	
	**Laboratory result & detailed information** **Breast CT and IHC:** breast lump, high metastatic breast sarcoma; **Thyroid US:** 5.6-cm hypoechogenic nodule and anecogenic areas fine-needle biopsy and was diagnosed with follicular lesion of undetermined significance (FLUS) (Bethesda category III); **Genetic test:** a change in heterozygote, pathogenic variant c.405dupA (p. (Cys136Metfs * 44) in the *PTEN* gene										
India (2015) [43]	41	F		Lesion on neck, axillae, forehead		Macrocephaly, mucocutaneous lesions, oral papules, acral keratosis		Thyroid lesions		**Family history:** NA	Ultrasound abdomen & pelvis, chest X-ray & ECG, brain MRI, GI endoscopy, colonoscopy, mammography
										**Surgical history:** Thyroidectomy	
										**Medical observations:** Syndactyly and polydactyly, cobblestone tongue with coalesced papules	
	**Laboratory result & detailed information** Imaging studies (ultrasound abdomen and pelvis, chest x-ray, ECG, MRI brain and echocardiography) and upper GI endoscopy, colonoscopy and mammography were normal										
USA (2017) [44]	64	F		Painful gum & red painful palate		Breast carcinoma, mucocutaneous lesions, oral papules, acral keratosis		NA		**Family history:** NA	Extra & intra oral examination, IHC
										**Surgical history:** NA	
										**Medical observations:** Malignancy of GI tract, anemia, asthma, breast cancer	
	**Laboratory result & detailed information** **Intraoral examination:** papules, pebbles on the case of the tongue and maxillary; **IHC:** Superficial keratinized squamous epithelium overlying a mass of dense fibrous connective tissue composed of interlacing bundles of collagen fibers interspersed by fibroblasts and blood vessels										
Japan (2014) [45]	37	M		Eyeball movement and gait disturbance		Mucocutaneous lesions, oral papules, acral keratosis		Intellectual disability, Vascular anomalies		**Family history:** CS in his mother with breast, thyroid & uterus cancers	Brain CT & MRI, DSA, IHC, genetic test
										**Surgical history:** Intracranial surgery	
										**Medical observations: H**emiparesis, left facial & right abducens nerve palsy, right Horner syndrome	
	**Laboratory result & detailed information** **Brain CT & MRI:** hematoma diameter 27 mm & circular area of heterogeneous intensity in the pons; **DSA:** no abnormal vessels & no tumour staining; **IHC:** dura mater contained abnormal vessels & ectatic cortical veins presented in the arachnoid space; **Genetic test:** *PTEN* mutation										
Italy (2018) [46]	43	M		Lesion localized on the gingiva		Breast carcinoma, thyroid carcinoma, mucocutaneous lesions, oral papules, acral keratosis, trichilemmoma		NA		**Family history:** Mother & sister suffered from CS and died from breast cancer	IHC, panoramic radiography
										**Surgical history:** Mastectomy**,** thyroidectomy	
										**Medical observations:** Breast & Thyroid carcinoma, periodontal disease	
	**Laboratory result & detailed information** **IHC:** hyper plastic epidermoid covering and parakeratosis on the surface; Since the diagnosis of the disease had already been made, there was no information about the diagnostic methods										
Japan (2020) [47]	31	F		Abnormal genital bleeding		Breast carcinoma, Macrocephaly, Endometriosis carcinoma		NA		**Family history:** CS in mother	Pelvic US, abdominopelvic CT, IHC, genetic test
										**Surgical history:** D&C, hysterectomy, salpingo-oophorectomy	
										**Medical observations:** Obesity, mass in the left ovary, APAM of endometrium and ovarian metastasis	
	**Laboratory result & Detailed information:** **Pelvic US:** thickening of the endometrium and a cystic mass in the left ovary; **Abdominopelvic CT:** endometrial thickening and a 5-cm cystic mass in the left adnexal region; **IHC:** grade 1 endometriosis carcinoma component presenting cribriform glands; **Genetic test:** Pathogenic variants of *PTEN* (c.C1003T, p.R335X) in both the patient and her mother										
Spain (2019) [48]	52	F		Multiple warts of hands & feet		Endometrial carcinoma, Mucocutaneous lesions, oral papules, acral keratosis, trichilemmoma		Thyroid lesions, Fibroma		**Family history:** Negative	GI endoscopy, genetic test
										**Surgical history:** hysterectomy, thyroidectomy	
										**Medical observations:** Bilateral cholesteatoma, trigeminal schwannoma, uterine fibroids, finger fibroma, tracheal fibrotic nodules, trichilemmoma, oral fibroma	
	**Laboratory result & Detailed information:** **GI endoscopy:** glycogenic acanthosis and ten colorectal inflammatory and hyperplastic polyps; **Genetic test:**a heterozygous mutation = a four-nucleotide deletion (c.510_513del) resulting in a premature stop codon (p.Ser170Argfs*12)										
Lebanon (2017) [49]	55	F		Left axillary sentinel node		Breast carcinoma, Macrocephaly, Endometrial, carcinoma		Lipomas, Fibroma		**Family history:** Breast cancer in one of siblings	Axilla &Brain MRI, IHC, genetic test
										**Surgical history:** Partial mastectomy	
										**Medical observations:** Papillary sclerosing lesion, DCIS, gangliocytoma, neurofibroma meningioma, extracranial meningothelial proliferation	
	**Laboratory result & Detailed information:** **Axilla MRI:** not demonstrate any suspicious lymph nodes; **IHC**: high grade ductal carcinoma in situ; **Brian MRI:** no lesions suggestive of meningioma; **Genetic test:** *PTEN* c.209 + 5G > A alteration confirming the clinical impression of CS										
Japan (2019) [15]	39	F		Lower abdominal pain & melena		Macrocephaly, Endometriosis carcinoma		Thyroid lesions, Lipoma, Fibroma, GI hamartomas		**Family history:** Breast cancer in mother and cutaneous papilloma in father	Colonoscopy, IHC, GI endoscopy, MRI of pelvic, CT, FDG-PET
										**Surgical history:** Pelvic & para-aortic lymphadenectomy, hysterectomy, salpingo-oophorectomy, omentectomy	
										**Medical observations:** Macrocephaly**,** breast fibroma, ovarian tumour, endometriosis cancer, colorectal polyposis lesions	
	**Laboratory result & Detailed information:** **Colonoscopy:** colorectal polyposis lesions; **IHC:** hamartoma polyps and ectopic endometrial implants; **GI endoscopy:** multiple esophageal papillomas and glycogenic acanthosis; **MRI:** gluteal subcutaneous lipoma **CT:** heterogeneously enhanced mass, 9 cm; **FDG-PET:** abnormal uptake by the ovarian tumour (SUVmax: 8.33)										
Japan (2015) [50]	75	M		Hoarseness & dysphagia		Mucocutaneous lesions, oral papules		Thyroid lesions, Vascular anomalies		**Family history:** NA	MRI, histology & IHC, GI endoscopic, genetic test
										**Surgical history:** NA	
										**Medical observations:** Lung adenocarcinoma, cerebellar tumour, GI polyposis, pneumonia	
	**Laboratory result & Detailed information:** **MRI:** cerebellar dysplastic gangliocytoma; **IHC**: lung adenocarcinoma (T4N2M1b, Stage IV); **GI endoscopic:** Gastrointestinal polyposis; **Genetic test:** *PTEN* gene mutation, a point mutation (*TGT* to *CGT*) at exon 5 in codon 136 was detected in his serum										
USA (2016) [51]	51	M		Jaundice & abdominal pain		Macrocephaly, thyroid carcinoma, mucocutaneous lesions, oral papules, acral keratosis, trichilemmoma		Lipomas, Thyroid lesions		**Family history:** Macrocephaly	Chest, abdomen & pelvis CT& PET-CT, pathological evaluation, IHC, Endoscopic sonography, genetic test
										**Surgical history:** Thyroidectomy	
										**Medical observations:** Macrocephaly, thyroid carcinoma, mucocutaneous, multiple intestinal polyps	
	**Laboratory result & Detailed information:** **CT:** large (9 × 8 × 4 cm) ill-defined mass of the head and neck of the pancreas, invading the celiac axis; encasing the celiac artery, superior mesenteric, and splenic arteries and veins; and compressing the portal vein confluence; **PET-CT:** large central lesion in the abdomen; **IHC& pathological evaluation:** cytoplasmic staining of tumour cells, grade 1 NET with a Ki-67 < 2% and involved margins; **Endoscopic sonography:** stage uT4N1Mx determined **Genetic test:** nonsense mutation c.697C3T (p.R233*) causing a premature stop codon in exon 7										
Japan (2018) [16]	46	M		Fresh blood in his stool & shortness of breath		Mucocutaneous lesions, oral papules, acral keratoses		GI hamartomas, Thyroid lesions, Fibroma, Vascular anomalies		**Family history:** Breast cancer in his mother	Neck US, GI endoscopy, pathophysiological examination, CT angiography
										**Surgical history:** Gastrectomy	
										**Medical observations:** Duodenal ulcer, anemia, adenomatous goiter, vascular anomalies	
	**Laboratory result & Detailed information:** **US:** adenomatous goiter in the thyroid gland; **GI endoscopy:** numerous polyposis lesions; **CT angiography:** vascular malformation in the wall of the sigmoid colon; **Pathological examination:** vascular malformations expanded from the submucosal layer to the mesocolon										
USA (2019) [52]	50	M		Vomiting, vertigo and balance difficulties		LDD, Macrocephaly		Thyroid lesions		**Family history:** CS	Brain MRI, IHC
										**Surgical history:** Thyroidectomy, right cerebellar LDD resection, Parathyroidectomy	
										**Medical observations:** multinodular goiter; gangliocytoma, abducens cranial nerve palsy; altered mental status, Parathyroid adenoma	
	**Laboratory result & Detailed information:** **MRI:** cerebellar tumour and progression of the left cerebellar gangliocytoma **IHC:** expansion of the cerebellar folia, with near complete replacement of the small neurons of the internal granular layers by larger, ganglionic neurons										
USA (2016) [53]	26	F		Painful perianal lesion		Macrocephaly, Mucocutaneous lesions, trichilemmoma		Thyroid lesion, Lipoma, Colon cancer		**Family history:** NA	Histopathologic examination, IHC, breast US
										**Surgical history:** hemicolectomy, Subtotal colectomy, Breast lumpectomy	
										**Medical observations:** Ganglioneuromas, focal intramucosal adenocarcinoma of the colon, proliferative glandular lesion, breast lesions	
	**Laboratory result & Detailed information:** **Histopathological examination:** a circumscribed, lobulated, proliferative glandular lesion with areas of expanded fibromyxoid stroma and epithelial hyperplasia with apocrine and columnar cell changes, arranged in a papillary, micropapillary and cribriform architecture; **IHC:** loss of *PTEN *expression in the epithelial component of the lesion.										
USA (2020) [54]	35	F		Forgetfulness and fatigue		LDD		NA		**Family history:** NA	Cerebral MRI, IHC
										**Surgical history:** Craniotomy for resection of meningioma, stereotactic radiosurgery	
										**Medical observations:** Meningiomas, dysplastic gangliocytoma	
	**Laboratory result & Detailed information:** **MRI:** lesion in the cerebellum suggesting dysplastic gangliocytoma; **IHC:** EMA and vimentin, consistent with World Health Organization (WHO) grade I meningioma margin. *The patient had already been diagnosed with the CS*										
Italy (2012) [55]	14	M		Epileptic seizures		Macrocephaly, Mucocutaneous lesions, oral papules, acral keratosis		Autism, Intellectual disability		**Family history:** Macrocephaly, dysthyroidism, GI cancers, autism, and behavioral and psychiatric disorders	Cerebral MRI, ECG, IHC, genetic test, laboratory test
										**Surgical history:** Nephrectomy	
										**Medical observations:** Autism, macrocephaly	
	**Laboratory result & Detailed information** **MRI:** right periventricular hyperintensity probably due to a remote hypoxic-ischemic insult; **ECG:** focal spike-wave complexes over the left temporal regions. **IHC:** focal prominent epidermal hyperplasia that at its periphery is demarcated by elongated rete ridges; **Genetic test:** an exon 2 deletion was detected; **Laboratory test:** moderate increase of thyrotrophin-stimulating hormone and free triiodothyronine and uncertain values for anti-endomysial antibodies with positivity for human leukocyte antigen (HLA) haplotypes (HLADQ2 and HLA-DQ8) associated with celiac disease										
USA (2018) [56]	57	F		Progressive weakness &urinary incontinence		Breast carcinoma, Macrocephaly		RCC, Lipomas, Vascular anomalies		**Family history:** Positive of cancers	MRI, spinal angiography, genetic test
										**Surgical history:** Nephrectomy	
										**Medical observations:** DCIS, multiple malignancies, squamous cell carcinoma of scalp, diabetes, multiple benign colon polyps, longitudinally extensive myelopathy, epidural lesions with bony erosion, thoracolumbar spinal cord lesion	
	**Laboratory result & Detailed information** **MRI:** longitudinally extensive myelopathy; **Spinal angiography:** 2 SEAVFs; **Genetic test:** A deleterious mutation (209 + 4_209 + 7delAGTA) was found in intron 3 of the *PTEN*										
Korea (2021) [57]	23	F		NA		Endometrial carcinoma, Mucocutaneous lesions		Thyroid lesion, Vascular anomalies		**Family history:** NA	Colonoscopy, endoscopy, genetic test
										**Surgical history:** Bariatric salpingectomy, Surgery for congenital vascular malformation of the leg, surgery for atypical ductal hyperplasia in the left breast & intraductal papilloma in the right breast, thyroid lobectomy & right central neck dissection pelvic lymph node dissection, left thyroidectomy, total hysterectomy, laparoscopic sleeve gastrectomy	
										**Medical observations:** Obesity & diabetes; before and after surgery, changes in HOMA-IR and HOMA-B; Cystic lesion in thyroid gland, multiple colon polyps, small bowel polyps, metastatic papillary thyroid cancer to the lung	
	**Laboratory result & Detailed information** **Colonoscopy:** multiple colon polyps (biopsy: lymphoidpolyps) were detected; **Capsule endoscopy**: scattered small bowel polyps; **Genetic test:** variant in which cytosine, the 289th base of the *PTEN* gene, was changed to thymine (c.289C > T). Consequently, glutamine, the 97th amino acid, was replaced with a stop codon (p.Gln97*)										
Italy (2014) [58]	26	M		asymptomatic keratotic lesions of the maxillary & mandibular gingiva		Mucocutaneous lesions, oral papules, acral keratosis		Fibroma		**Family history:** NA	Laboratory tests, IHC, GI endoscopy, genetic test
										**Surgical history:** NA	
										**Medical observations:** celiac disease, papillomatous lesions of the penis, hands & plantar skin, gastric polyposis lesions	
	**Laboratory result & Detailed information** **Hematology and chemistry:** hematologic tests, including BS, Na, K, Ca, AST, ALP, LDH, Cr, HCT, RBC, HB, MCV, Plt, WBC, HIV, hepatitis B, C; All Laboratory test values were normal; **IHC:** proliferation of multiple benign fibromas with overlying hyperkeratosis; **GI endoscopy:** gastric polyposis lesions; **Genetic test:** germline mutation in exon 8										
Brazil (2012) [59]	24	F		asymptomatic lesions in the oral cavity		Macrocephaly, Mucocutaneous lesions, oral papules		Thyroid lesion		**Family history:** ovarian & breast cancer in her mother	Histopathologic evaluation, endoscopy, Skull x-ray
										**Surgical history:** thyroidectomy	
										**Medical observations:** chronic urticaria with symptomatic dermographism, delayed motor and psychiatric development, hypotonia, macrocephaly, pectus excavatum, scoliosis, dolichocephaly and adenoid facies; polyposis in the GI tract, nodularhyperplasia of the thyroid	
	**Laboratory result & Detailed information** **Hematology and chemistry**; including Ca, ALP and AST and urinalysis were normal; **Histopathologic analysis:** nodular hyperplasia of the thyroid; **Endoscopy:** polyposis in the GI tract										
Japan (2018) [60]	36	M		Facial seizure during sleep		Macrocephaly, Mucocutaneous lesions, oral papules		NA		**Family history**: hepatocellular carcinoma in his father	Brain MRI, CT-scan GI endoscopy, genetic test, PCR
										**Surgical history:** NA	
										**Medical observations:** Macrocephaly**,** facial seizure	
	**Laboratory result & Detailed information** **Blood tests:** for autoantibodies, amino acids, lactate, pyruvate, pituitary hormone, tumour markers, and infectious agents showed no abnormalities; **Brain MRI:** focal cortical dysplasia; **CT:** hypometabolism of cortical dysplasia; **GI endoscopy:** multiple polyps **Genetic test:** heterozygous deletion in exon 5 (c.486delC)										
Portugal (2016) [61]	36	F		Headache & visual blurring bilaterally		Thyroid cancer, mucocutaneous lesions, acral keratosis, LDD		NA		**Family history**: breast carcinoma in her mother & sister	CT, MRI, OCT, genetic test
										**Surgical history:** thyroidectomy	
										**Medical observations:** FDB	
	**Laboratory result & Detailed information** **CT:** unilateral cerebellar mass in the right hemisphere; **MRI:** right cerebellar mass with secondary hypertrophy of the cerebellar folia with a striated or tigroid pattern characteristic of LDD; **OCT**: thickening of peripapillary retinal nerve fiber layer; **FA:** leakage from the optic discs and confirmed papilledema; **Genetic test:** *PTEN* mutation c.493G > T										
USA (2018) [62]	32	F		Osteosarcoma and its screening process		NA		FDB		**Family history:** Bladder cancer in her maternal grandfather, ovarian cancer in her maternal grandmother; brain, breast, colon, thyroid cancer in her mother; a soft-tissue mass in her brother	Genetic test, colonoscopy, endoscopy
										**Surgical history:** Surgery for osteosarcoma of femur, prophylactic bilateral mastectomy	
										**Medical observations:** Osteosarcoma of her left femur, right benign breast mass	
	**Laboratory result & Detailed information** **Genetic test:** non-synonymous c.17_18delAA frameshift mutation in exon 1 of *PTEN* and a c.116G > T (p.R39L) missense mutation of serine/threonine kinase 11(STK11) of unknown significance; **Colonoscopy & Pathological examination**: 75–100 polyp and ganglioneuromas in splenic flexure, descending and sigmoid colon										
Portugal (2017) [63]	53	M		Fibromas on the trunk and hyperkeratotic lesions on the hands		Mucocutaneous lesions, oral papules, acral keratosis, trichilemmoma, thyroid carcinoma, penile melanosis, macrocephaly		Fibroma, Lipomas, GI hamartomas		**Family history:** Negative	Genetic test, laboratory tests
										**Surgical history:** Thyroidectomy	
										**Medical observations:** Malignant thyroid disease, intestinal polyposis, papillomatosis of the oral cavity	
	**Laboratory result & Detailed information** **Genetic test:** *PTEN* mutation; **Laboratory test & renal ultrasound**: no significant changes										
Japan (2018) [64]	65	F		Lesions on skin		Thyroid carcinoma, mucocutaneous lesions, oral papules, acral keratoses		FDB, Vascular anomalies		**Family history:** NA	Mammography, genetic test, Ileocolonoscopy
										**Surgical history:**	
										**Medical observations:** Malignant thyroid disease, Intestinal polyps, oral cavity papillomatosis	
	**Laboratory result & Detailed information** **Mammography**: carcinoma of the left breast; **Genetic test:** a heterozygous c.1003C > T (p.R335X) mutation of the phosphatase and tensing homolog (*PTEN*) gene; **Ileocolonoscopy:** hemispherical or drumstick-shaped multiple polyps in the terminal ileum										
USA (2020) [65]	56	F		Diplopia Headaches		LDD		RCC		**Family history:** NA	MRI
										**Surgical history:** NA	
										**Medical observations:** Hashimoto’s thyroiditis, RCC	
	**Laboratory result & Detailed information** **MRI:** non-enhancing cerebellar lesion, the patient was previously diagnosed with CD and we can exclude it										
USA (2015) [66]	31	M		Lump in his right breast		Macrocephaly, Breast carcinoma		Thyroid lesion		**Family history:** Hodgkin lymphoma in great grandparent, leukemia in his aunt, breast cancer and penis cancer in great grandparents	Chest CT, thyroid US, abdominal CT, genetic test
										**Surgical history:** Bilateral mastectomies	
										**Medical observations:** lymphadenopathy	
	**Laboratory result & Detailed information** **Chest CT:** mediastinal mass; **Genetic test:** heterozygous missense variant (c.103A > G; p.Met35Val) was observed in the *PTEN* gene; **Thyroid US:** multiple nodules; **Colonoscopy:** sessile polyps										
Singapore (2021) [67]	48	F		Headache and dizziness		Breast carcinoma, LDD		GI hamartoma		**Family history:**	Brain CT, MRI, IHC
										**Surgical history:** resection of the tumor of brain	
										**Medical observations:** hemangioma, breast cancer, hamartomatous polyps	
	**Laboratory result & Detailed information** **Brain CT**: large hypodense lesion at the right cerebellum with resulting hydrocephalus; **MRI:** tiger-striped pattern of cerebellar folia in the right cerebellum; **IHC:** ganglion cells were positive for synaptophysin and neurofilament										
Korea (2012) [68]	29	F		Dyspepsia		Mucocutaneous lesions, oral papules, acral keratosis, trichilemmomas, thyroid carcinoma		GI hamartoma Thyroid lesion		**Family history:**	EGD, colonoscopy, genetic test
										**Surgical history:** left lobectomy	
										**Medical observations:**	
	**Laboratory result & Detailed information** **EGD:** gastric, duodenal polyps and esophageal acanthosis; **colonoscopy:** hamartomatous polyps in the rectum and oral mucosal papillomatosis **Genetic test:** heterozygous transition of C to A at nucleotide 633 in exon 6 (NM_000314.4 c.633C > A), which is a nonsense mutation, making a stop codon (p.Cys211*)										
Iran (2010) [19]	16	F		Subtle signs of CS		Mucocutaneous lesions, acral keratosis, trichilemmomas		FDB		**Family history:** NA	PCR-SSCP, HMA, genetic test
										**Surgical history:** Mastectomy	
										**Medical observations:** FDB	
	**Laboratory result & Detailed information** **PCR-SSCP:** all identified mutations were present in exon 5; **HMA:** heteroduplex bands with mobility shifts in lanes 2,5&6 are mutant samples **Genetic test**: nucleic acid alteration = c.341 T > G										
UK (2015) [17]	48	M		Dysphagia Right hearing loss and facial weakness		LDD		Thyroid lesion		**Family history:** LDD in his nephew	IHC, MRI, CT-scan
										**Surgical history:** brain& neck resection mass	
										**Medical observations:** nasal polyps, high-grade neoplasm involving the facial nerve, deafness	
	**Laboratory result & Detailed information** **IHC:** for neurofilament protein showed residual nerve fibers infiltrated by the tumour; **MRI and CT:** brain and neck showed a mass (4.2*2.9 cm) at the cerebellopontine angle; **Genetic test:** Nucleic acid alteration = c.1003C > T, amino acid alteration = p.(Arg335Ter), the genetic testing of the proband’s offspring identified the same mutation										
Italy (2021) [69]	46		M		Abdominal pain		Mucocutaneous lesions, oral papules, acral keratoses, macrocephaly		GI hamartoma	**Family history:** Colon and breast cancer in his mother	Colonoscopy, IHC, EGD, genetic test
										**Surgical history:** NA	
										**Medical observations:** Macrocephaly; polypoid lesions in GI	
	38		F		Abdominal pain		Thyroid carcinoma, mucocutaneous lesions, oral papules, acral keratosis, trichilemmoma		FDB, Fibroma	**Family history:** Negative	Endoscopy, colonoscopy, IHC, EGD, uterus US, genetic test
										**Surgical history:** Thyroidectomy & mastectomy	
										**Medical observations:** Thyroid carcinoma, FDB with lobular carcinoma in situ, uterine fibroids and squamous polyp of uterine cervix	
	**Laboratory result & detailed information** **Case 1 (M). colonoscopy:** more than 20 sessile and pedunculated polyposis lesions throughout the entire colon and the terminal ileum; **IHC:** large cistically dilated tortuous intestinal glands surrounded by stroma; **EGD:** multiple small carpeting lesions; **Genetic test:** heterozygous variant c.306del, p.Lys102Asnfs*11 (NM_000314.8) was detected in the exon 5 of the *PTEN* gene **Case 2 (F): Endoscopy:** Gastrointestinal polyposis lesions; **Colonoscopy:** more than 20 sessile polyps involving the entire colon; **IHC:** simultaneous presence of enlarged lymphoid follicles and hyperplastic polyps; **EGD:** numerous millimetric sessile lesions covering all the esophageal mucosa and gastric mucosa; **Genetic test:** heterozygous variant c.253 + 1G > A; p.? (NM_000314.8) (rs587776667)										
Iran (2022) [This study]	39	F		Rectorrhagia refractory dyspepsia		Breast carcinoma		Thyroid lesion		**Family history:** Gastric cancer in her father & breast cancer in her cousin	Endoscopy, colonoscopy, laboratory test, genetic test, breast UC, histopathological examination, endometrial biopsy, abdominal CT scan, thyroid sonography
										**Surgical history:** Partial mastectomy	
										**Medical observations:** Polyps in stomach, duodenum, rectosigmoid	
	**Laboratory result & Detailed information** **Hematology:** WBC = 2.2 × 10^3^/ μL, RBC = 4.141 × 10^6^/ μL, HGB = 9.2 g/dl, PLT = 443 × 10^3^/ μL; **Chemistry:**T3, T4, and TSH were within thenormal limits; **Genetic test:** RefSeq = NM_00314.7, Nucleic acid alteration = c.697C > T, Amino acid alteration = (p.Arg233Ter) rs121909219, Zigosity = Heterozygous, Ch. Location = Chr10 Mutation: Pathogenic										

*APAM* Atypical polypoid adenomyoma, *CT* Computerized tomography, *CCA* Clear cell acanthoma, *D&C* Dilatation and curettage, *DSA* Digital subtraction angiography, *ERCP* Endoscopic retrograde cholangio-pancreatography, *ECG* Electroencephalography, *FDG-PET* F-fluorodeoxyglucose-positron emission tomography, *FA* Fluorescein angiography, *FDP* Familial defective apolipoprotein, *FDB* Fibrocystic disease of the breast, *GI* Gastrointestinal, *HMA* Heteroduplex mobility analysis, *IHC* Immunohistochemistry, *LDD* Lhermitte-Duclos disease, *MRI* Magnetic resonance imaging, *PCR* Polymerase chain reaction, *PET* Positron emission tomography, *RCC* Renal cell carcinoma, *SSCP* Single strand conformation polymorphism, *SEAVF* Spinal epidural arteriovenous fistulas, *SLA* Sebaceous lymphadenoma, *US* Ultrasonography

^a^Shows the chief complains of each patient

^b^Shows the major criteria considered to diagnose PHTS

^c^Shows the major criteria considered to diagnose PHTS

^d^Shows the family, surgical and medical history of each patient

^e^Shows the diagnostic measures applied for each patient

^f^Shows the detailed results of each diagnostic measures performed

## Discussion

In this report, we described the features of PHTS in an Iranian female patient. Our initial examination revealed the presence of 50–100 rectosigmoid polyps, which prompted us to apply WES to confirm hereditary colorectal polyposis. Intriguingly, despite the patient’s lack of skin or clinical symptoms of the condition, the WES results revealed a heterozygous c.697C > T (p.Arg233Ter) pathogenic variant of the *PTEN* gene that is linked with autosomal dominant PHTS. However, the patient’s family history was found to be significant. Previous studies indicate that PHTS have a family history in one-third of patients [70, 71]. The father and a cousin of our patient had cancer, while our literature review revealed a family history of cancer for 42% of patients with PHTS. Therefore, it is important to screen family members and obtain a thorough family history to identify additional cases of PHTS as early as possible.

Acral keratosis, trichilemmomas, and mucocutaneous papillomata are pathognomonic features of PHTS [72]. Literature reports reveal that cutaneous lesions manifest as trichilemmomas and acral keratosis, with pits on the palms, lips and soles in around 70% of PHTS patients. However, our case lacked these lesions.

PHTS is associated with a high prevalence of breast, thyroid, and endometrial neoplasias, which are the primary complications of the disease [34, 73]. The lifetime risk of developing breast cancer in women with PHTS ranges from 54.3 to 75.8% [34]. Hence, women with PHTS are recommended to self-examine their breasts and undergo regular mammography [13]. In the present case, mammography facilitated the early detection of breast cancer. Preventative measures such as bilateral mastectomy are advised for PHTS patients with extensive fibrocystic breast disease or breast cancer [74].

Compared to previously reported rates [2, 9, 34, 73, 75–77], recent literature suggests that PHTS patients may have an even higher risk of developing breast cancer (up to 80%) and endometrial cancer (up to 28%). In addition, these patients may develop a significant number of benign thyroid lesions (up to 60%) and thyroid cancer (over 10%) [34, 78]. Genetic and molecular studies of PHTS have revealed the presence of pathogenic variants in the *PTEN/MMAC1* gene, located on chromosome 10 at position q 22–23, which is implicated in breast and thyroid cancer [73, 79–82]. While the clinical and laboratory diagnostic criteria serve as the foundation for diagnosis [9], molecular genetic testing can be used to identify these pathogenic variants [55, 83]. However, recent prospective studies suggest that the prevalence of germline pathogenic variants in *PTEN* can be estimated at only 25% of patients with this condition, which is lower than previously thought [2, 84]. We applied both approaches and obtained positive results for our patient, who was diagnosed with breast cancer, multiple thyroid nodules, and more than the 50–100 range of rectosigmoid polyps in addition to a significant family cancer history.

Patients with PHTS may also develop tumours in various parts of the body, including the gastrointestinal or genitourinary tract, or the brain [85, 86]. Gastrointestinal polyposis is a common symptom that affects any part of the digestive system [20, 87, 88]. It is essential to recommend earlier endoscopic screening due to the high frequency of colon polyps in PHTS, estimated to be between 65.6 and 93% [38]. However, the risks and benefits of early intervention should be carefully weighed against the financial expenses and health risks associated with increased endoscopic surveillance [38].

Innella et al. [69] reported two cases of PHTS referred for genetic testing due to endoscopic findings of multiple colorectal polyps, which was similar to our case. While our literature review showed gastrointestinal polyps to be common in PHTS patients, mucocutaneous lesions are the most common diagnostic criteria. Therefore, all the various manifestations of PHTS among these patients should be carefully considered. Colorectal screening, starting at age 35–40 years [85, 89], is recommended for those with *PTEN* pathogenic variants.

Table 3 demonstrates that both the upper and lower gastrointestinal tracts are frequently involved in PHTS patients, including our case which revealed numerous small rectosigmoid, gastric and duodenal polyps. These findings suggest that colon polyposis is an under-reported characteristic in PHTS guidelines. Furthermore, evidence supports the elevated risk of colorectal cancer in PHTS patients [34, 76, 85, 89, 90]. However, the association between PHTS and gastrointestinal malignancies is still a controversial subject [40, 78, 91–95].

The dysplastic, cerebral ganglion cell tumour LDD is a significant pathological diagnostic criterion for PHTS [40, 74, 96]. To rule out its potential presence, it is recommended that patients presenting with headaches undergo MRI of the brain, as has been stated in multiple sources [61, 65, 67]. While ovarian tumours are a rare occurrence in PHTS, this possibility should not be overlooked as an ovarian dysgerminoma has been documented in a patient with PHTS [13].

To the best of our knowledge, this is the first reported case of PHTS detected through WES during a hereditary polyposis evaluation of rectosigmoid polyps in an Iranian patient. Although our review was not a systematic one, we found 43 case reports on PHTS. It is therefore probable that this affliction than though so far. Indeed, our search was limited to PubMed and we may well have missed additional cases reported in other databases. Although a language bias may exist with regard to our review, the English tongue is widely perceived as the universal language of science, and studies in medical sciences have not shown any systematic bias resulting from this restriction [97, 98].

## Conclusion

The detection of rectosigmoid polyps should prompt practitioners to consider genetic evaluation for hereditary colorectal polyposis and to also consider the possibility of PHTS and thus look for other associated manifestations of this syndrome. Importantly, the finding of pathogenic variants in the *PTEN* gene led to early screening for breast cancer with a positive outcome. The presence of > 50 polyps in the rectosigmoid, coupled with the absence of typical familial adenomatous polyposis or other forms of colon polyposis, warrants further studies to identify the PHTS phenotype. Early cancer detection through regular surveillance is critical for the management of PHTS and has been shown to improve overall survival. Thus, all PHTS patients should receive annual thyroid ultrasound scans and dermatologic evaluations, while women should receive annual mammograms and breast MRIs from age 30, along with annual transvaginal ultrasound investigations and blind suction endometrial biopsies.

## Data Availability

This paper contains all the necessary information for others to reproduce our findings, including the raw data used in the genetic evaluation (WES), which have been made available as a supplementary file [30]. These data can be used for research purposes with appropriate citation of this paper.
